# Prevention and Management of Dermatologic Adverse Events Associated With Tumor Treating Fields in Patients With Glioblastoma

**DOI:** 10.3389/fonc.2020.01045

**Published:** 2020-07-28

**Authors:** Mario E. Lacouture, Milan J. Anadkat, Matthew T. Ballo, Fabio Iwamoto, Suriya A. Jeyapalan, Renato V. La Rocca, Margaret Schwartz, Jennifer N. Serventi, Martin Glas

**Affiliations:** ^1^Memorial Sloan Kettering Cancer Center, New York, NY, United States; ^2^Division of Dermatology, Department of Internal Medicine, Washington University School of Medicine, St. Louis, MO, United States; ^3^Department of Radiation Oncology, West Cancer Center, Memphis, TN, United States; ^4^New York-Presbyterian/Columbia University Medical Center, New York, NY, United States; ^5^Department of Neurology, Tufts Medical Center, Boston, MA, United States; ^6^Department of Hematology-Oncology, Tufts Medical Center, Boston, MA, United States; ^7^Norton Cancer Institute, Norton Healthcare, Louisville, KY, United States; ^8^Northwestern Medicine, Chicago, IL, United States; ^9^University of Rochester Medical Center, Rochester, New York, NY, United States; ^10^Division of Clinical Neurooncology, Department of Neurology and West German Cancer Center, German Cancer Consortium, Partner Site Essen, University Hospital Essen, University Duisburg-Essen, Essen, Germany

**Keywords:** glioblastoma, safety, skin management, Tumor Treating Fields, TTFields, Optune

## Abstract

**Importance:** Tumor Treating Fields (TTFields) are an anti-mitotic treatment approved for treating newly diagnosed and recurrent glioblastoma, and mesothelioma. TTFields in glioblastoma comprise alternating electric fields (200 kHz) delivered continuously, ideally for ≥18 h/day, to the tumor bed via transducer arrays placed on the shaved scalp. When applied locoregionally to the tumor bed and combined with systemic temozolomide chemotherapy, TTFields improved overall survival vs. temozolomide alone in patients with newly diagnosed glioblastoma. Improved efficacy outcomes with TTFields were demonstrated, while maintaining a well-tolerated and manageable safety profile. The most commonly-reported TTFields–associated adverse events (AEs) are beneath-array dermatologic events. Since survival benefit from TTFields increases with duration-of-use, prevention and management of skin AEs are critical to maximize adherence. This paper describes TTFields-associated dermatological AEs and recommends prevention and management strategies based on clinical trial evidence and real-world clinical experience.

**Observations:** TTFields–associated skin reactions include contact dermatitis (irritant/allergic), hyperhidrosis, xerosis or pruritus, and more rarely, skin erosions/ulcers and infections. Skin AEs may be prevented through skin-care and shifting (~2 cm) of array position during changes. TTFields–related skin AE management should be based on clinical phenotype and severity. Depending on diagnosis, recommended treatments include antibiotics, skin barrier films, moisturizers, topical corticosteroids, and antiperspirants. Water-based lotions, soaps, foams, and solutions with minimal impact on electrical impedance are preferred with TTFields use over petroleum-based ointments, which increase impedance.

**Conclusions:** Early identification, prophylactic measures, and symptomatic skin AE management help patients maximize TTFields usage, while maintaining quality-of-life and optimizing therapeutic benefit.

**Implications for practice:** TTFields confer a survival benefit in patients with glioblastoma that correlates positively with duration of daily use. Skin events (rash) are the primary treatment-related AE that can limit duration of use. The recommendations described here will help healthcare professionals to recognize, prevent, and manage dermatologic AEs associated with TTFields treatment. These recommendations may improve cutaneous health and support adherence to therapy, both of which would maximize treatment outcomes.

## Introduction

### Glioblastoma

Glioblastoma (GBM) is the most common and aggressive primary brain tumor (1). It is classified as a grade IV tumor (2), and median survival ranges from 14.6 to 16.8 months with standard therapies (3–6). The 5-year survival rate is estimated at 6.8% in the United States (US) (1), with rates up to 9.8% reported in clinical trials (3). Treatment is difficult because tumor margins are hard to visualize, resection may damage vital brain functions (7), and the blood–brain barrier limits entry of systemic therapies (8). In addition, there is variation in the genetic and molecular features of tumors, as well as a high degree of inter- and intra-tumor heterogeneity (9). For these reasons, standard-of-care therapy for GBM has been limited until recently, consisting only of maximal surgical resection followed by radiotherapy (60 Gy in 2.0 Gy fractions) plus concurrent temozolomide (TMZ) chemotherapy (75 mg/m^2^ daily), followed by adjuvant TMZ (150–200 mg/m^2^ on a 5-day schedule every 28 days) (10). The most common adverse events (AEs) related to concurrent radiotherapy and TMZ include fatigue (33%), grade 3/4 hematologic toxic effects including neutropenia and thrombocytopenia (7%), thromboembolic events (4%), and severe infections (3%) (10). GBM treatment continues to be challenging, since recurrence is almost inevitable despite treatment (10, 11).

### Tumor Treating Fields

**Figure 1:**
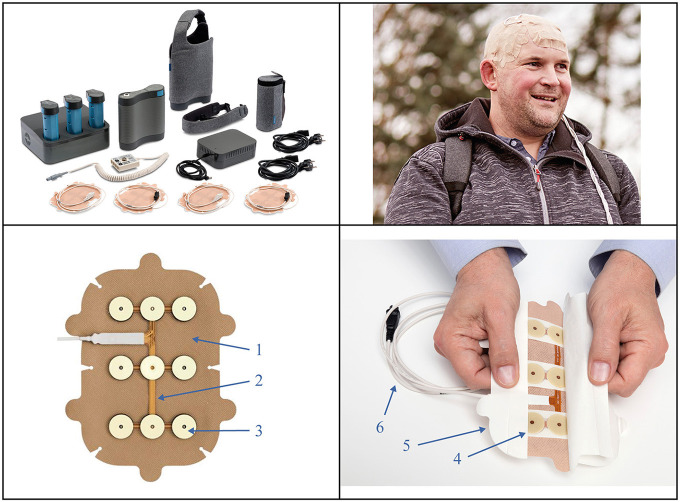
The Tumor Treating Fields (TTFields) device and transducer arrays. ***Top left panel:*** second generation (Gen 2) battery-operated field generator device, portable battery packs, plug-in power supply, tan transducer arrays, connection cables and box, and carrying case. This results in an increased operational efficiency and improved patient experience. ***Top right panel:*** Shows a patient* with glioblastoma during therapy, wearing the tan transducer arrays on his scalp. ***Bottom panels: 1***. A hypoallergenic cover tape holds tan arrays in place on the scalp. ***2***. Transducer arrays deliver low intensity, intermediate frequency (200 kHz) alternating electric fields and monitor the temperature of the scalp. ***3***. Conductive hydrogel layers *(top)* ensure separation between the arrays and skin, and the ceramic disks *(beneath)* transmit TTFields without direct contact with the skin. ***4***. Mid-pads mechanically stabilize the gel over the arrays. ***5***. An overlapping liner covers the gel and cover tape. ***6***. A cable connects array to the connection box. *Permission for global image use was obtained from the patient.

Tumor Treating Fields (TTFields; Optune®, Novocure Inc) are a unique, non-invasive, antitumor treatment modality that delivers low-intensity, alternating electric fields (200 kHz) locoregionally to tumor beds in the brain, through 2 pairs of orthogonally positioned transducer arrays affixed directly to the shaved scalp of patients with GBM (Figure 1) (12, 13). Alternating electric fields disrupt the rapid cell division of cancer cells and interfere with mitotic spindle microtubule formations, ultimately resulting in cancer cell death (12, 14). Additional mechanisms of action against cancer cells include apoptosis induction (15), DNA repair inhibition (16), DNA replication stress induction (17), migration and invasion impairment, angiogenesis suppression (18), autophagy upregulation (19), and immunogenic cell death (20). Moreover, in preclinical studies, TTFields reversibly increased tumor cell-specific membrane permeability (21) and had a transient effect on blood–brain barrier integrity and permeability, with the potential to deliver systemic therapies to the brain (22).

Each TTFields transducer array is composed of 9 biocompatible insulated ceramic disks covered by hydrogel on the skin-facing side and attached to a flexible adhesive bandage on the opposite (external) side (Figure 1). Arrays are supplied to patients in individual, presterilized packages to minimize cross-contamination and infection risk. The arrays are worn continuously for 3–4 days before removal for hygienic scalp skin care and reshaving of the scalp to ensure array contact with skin. Current standard practice during array changes is to wipe the skin with 70% ethanol (not recommended on irritated skin). Magnetic resonance imaging is used to guide the optimal array layout for each patient based on tumor location and extent (23). Each array has 8 temperature sensors (thermistors) that continuously monitor temperature; if the array temperature exceeds 41°C (105.8°F), the device will shut off and sound an alarm (24). Patients should use the device for ≥18 h/day on average, according to the EF-14 clinical trial design (6). Notably, increased TTFields usage is independently prognostic of improved survival in GBM, with a usage threshold of 50% and a maximum effect on survival with >90% usage (25, 26).

### TTFields in Clinical Practice

TTFields were approved for recurrent GBM in the European Union (EU) in 2009, the US in 2011, Japan in 2015 (27, 28), as well as recent approval in China in 2020 (with simultaneous approval as adjuvant therapy in newly diagnosed GBM), followed by approval in EU, US, and Japan as adjuvant therapy for newly diagnosed GBM (28). In the US, TTFields are approved as monotherapy for adult patients with recurrent supratentorial GBM and in combination with standard-of-care chemotherapy for newly diagnosed supratentorial GBM following surgery and radiotherapy. National Comprehensive Cancer Network (NCCN) guidelines recommend TTFields as a category 1 treatment in combination with TMZ after maximal safe resection and completion of radiotherapy in patients with newly diagnosed GBM (29), and the American Society of Clinical Oncology has recognized TTFields as a treatment that has advanced clinical cancer care (30).

Approvals were based on 2 pivotal, phase 3 clinical trials comparing TTFields therapy with active standard chemotherapy in patients with recurrent or newly diagnosed GBM (6, 31). In the EF-11 trial, TTFields demonstrated comparable overall survival (OS) to active chemotherapy in recurrent GBM, with a median OS of 6.6 vs. 6.0 months, respectively (hazard ratio [HR] = 0.86; 95% CI, 0.66–1.12; *P* = 0.27), though failed to demonstrate superiority, which was the primary objective (31). However, in the EF-14 trial, addition of TTFields to TMZ for newly diagnosed GBM resulted in a significantly improved median OS of 20.9 vs. 16.0 months with TMZ alone (HR = 0.63; 95% CI, 0.53–0.76; *P* < 0.001) (6). Significant survival benefits were reported irrespective of age, sex, performance status, and/or extent of resection. Long-term follow-up showed 5-year OS with TMZ alone was less than half that of TTFields plus TMZ (5 vs. 13%, respectively; *P* = 0.0037) (6).

In both phase 3 TTFields/GBM clinical trials (6, 31), TTFields-related AEs were mostly grade 1/2 (mild-to-moderate) in severity, manageable without substantial treatment breaks, and resolved completely after treatment was stopped. In the EF-11 trial of recurrent GBM, significantly more gastrointestinal, hematologic, and infectious AEs were observed with chemotherapy vs. TTFields (31). Moreover, the EF-14 trial of newly diagnosed GBM showed no significant increase in systemic AEs with TTFields when compared with TMZ alone (48 vs. 44%, respectively; *P* = 0.58) (6). Additionally, quality-of-life analyses of the EF-14 trial found no significant differences between treatment arms except for more itchy skin in TTFields-treated patients (32). Notably, 75% of patients used TTFields for the recommended ≥18 h/day, suggesting good tolerability (6).

Dermatologic conditions beneath the transducer arrays were the most commonly reported TTFields-related AEs in all clinical studies to date (33). In the EF-11 clinical trial, grade 1/2 skin AEs occurred in 16% of patients; all were manageable and reversible, with none resulting in discontinuation from the study (31). In the EF-14 trial, grade 1/2 skin AEs beneath the transducer arrays occurred in 52% of patients receiving TTFields plus TMZ compared with none receiving TMZ monotherapy (6). A retrospective analysis of real-world safety surveillance data from 7,408 patients with GBM treated with TTFields since 2011 confirmed clinical trial findings. Skin reactions were the most prevalent AE, occurring in 35% and 20% of patients with newly diagnosed or recurrent GBM, respectively (34). Furthermore, the post-marketing Patient Registry Dataset (PRiDe), including 457 patients with recurrent GBM treated with TTFields, reported a similar pattern of AEs, with 24.3% of patients reporting skin reactions beneath the transducer arrays, and 11.3% reporting heat sensations (local heat beneath the arrays; described as a warm sensation) (35). No new safety signals or unexpected AEs were reported. Furthermore, these published phase 3 TTFields/GBM clinical trial data and prior registry observations were consistent with findings from a recently published retrospective, real-world, global post-marketing surveillance analysis that evaluated safety of TTFields in patients with brain cancer in the real-world, clinical practice setting (36). This analysis reported on AEs from a large patient cohort who were TTFields-treated (*N* = 11, 029; largest dataset to date) as well as subgroups (region, diagnosis, and age). The majority of patients were diagnosed with ndGBM and rGBM (*n* = 10, 232; one of largest datasets of patients with GBM). Overall, TTFields treatment showed a favorable safety profile with no new safety signals in the total cohort and across subgroups and suggested feasibility in multiple subpopulations, including elderly patients. The most commonly reported array-related AE was localized, mild-to-moderate skin reactions and no treatment-related systemic effects were noted. These data have further confirmed the known safety and tolerability of TTFields for GBM.

In 2014, initial recommendations published on the characterization, prevention, and management of TTFields-associated dermatologic AEs concluded that prevention and timely management are crucial to maintaining patient quality-of-life, ensuring consistent use of TTFields, and ultimately maximizing clinical benefit (24). Adoption of TTFields therapy for GBM has subsequently increased considerably, allowing for symptom-based characterization, prevention, and management of dermatologic AEs associated with treatment in clinical practice. The objectives of the current recommendations are to better define the specific dermatologic AEs associated with TTFields and to summarize prophylactic and practical, treatment-based strategies for skin AE management based on evidence from clinical trials and real-world clinical experience.

## Characterization of Dermatologic Adverse events Associated With TTFields

TTFields-associated dermatologic AEs result from distinctive mechanical, thermal, chemical, and moisture-related stresses related to prolonged contact with transducer arrays and adhesive, which are applied sequentially to the same area of the skin (24). Thermal injury is unlikely, as the device shuts off and an alarm sounds if the array temperature exceeds 41°C, which is below the threshold for a thermal skin burn (24). Mechanical trauma from shaving and/or constant array pressure and reapplication may lead to epidermal loss with inflammation, which may be complicated by skin infections, erosions, and ulcerations, especially at the site of previous surgical scars. Irritant contact dermatitis may result from chemical irritation from the hydrogel or alcohol, and/or moisture (24). Predisposing factors for patients with GBM include prior radiation in the area, ongoing dexamethasone treatment, and combination treatment with chemotherapy (cytotoxic alkylating agents, such as TMZ), or targeted treatments (e.g., bevacizumab, an antiangiogenic agent, or mammalian target of rapamycin [mTOR]/mitogen-activated protein kinase [MEK] inhibitors). While bevacizumab is the only targeted therapy currently indicated for recurrent GBM, NCCN guidelines suggest compassionate use of other targeted therapies in patients with recurrent GBM and relevant mutations (29).

### Identification and Classification of Dermatologic AEs

Five types of dermatologic AEs have been identified with TTFields utilization, all of which are more likely to occur where the scalp makes contact with adhesive or hydrogel on the transducer arrays (Table 1) (24, 39).

1. **Hyperhidrosis** is excessive sweating of the scalp, which can be caused by multiple factors, including climate, physical activity, concomitant medications, and genetic predisposition (**Case 1**).
2. **Xerosis or pruritus** (alone or in combination) is caused by ambient humidity and temperature, concomitant medications, genetic predisposition, and skin water loss (**Case 2**).
3. **Contact dermatitis** is inflammation of the skin caused by irritant exposure. Exposure to chemical irritants elicits non-specific release of local inflammatory chemokines (irritant contact dermatitis), while exposure to exogenous allergens induces specific immunologic response mechanisms based on allergen sensitization (allergic contact dermatitis) (40). Dermatitis can manifest as erythema, edema, pruritus, or burning and scaling of the skin (**Case 3**).
4. **Skin erosions** are moist, circumscribed, depressed, secondary lesions that result from loss of a portion or all of the viable epidermis, but do not extend into the dermal layer (24). **Skin ulcers** are secondary lesions involving the epidermal and dermal layers, which may result in scarring (24). The base may be clean, necrotic, or contain granulation tissue. They may involve mild bleeding, pain, and/or burning (**Case 4**).
5. **Skin and soft tissue infections** are caused by damage to the skin barrier, resulting in an abundance of pathogenic microbes within the skin and supporting structures (37, 38). Pustules may contain a hair at the center (folliculitis), vary in size, and even coalesce (**Case 5**).

**Table 1:** Dermatologic scalp adverse event (AE) types, symptomatology, potential causes, and treatment recommendations (24, 37–40).

**Case 1: Tumor Treating Fields (TTFields) AE - hyperhidrosis**		
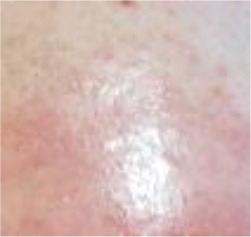	**Symptomatology**	**Potential Cause(s)**
	• Excessive sweating from scalp	• Genetic predisposition • Hot/humid climate • Intense activity • Medications
**Suggested Intervention(s)** • Treat with aluminum chloride antiperspirant or topical glycopyrrolate at every array exchange • Advise patients to avoid using ointments and medications that may cause sweating • Consider referral to a dermatologist for botulinum toxin injections		
**Case 2: TTFields AE – pruritus**		
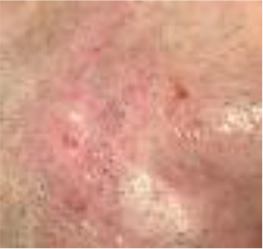	**Symptomatology**	**Potential Cause(s)**
	• Dry skin (xerosis) • Itchy skin (pruritus) • Flaky skin (dandruff)	• Genetic predisposition • Cold/dry climate • Loss of water/oil • Medications • May be related to contact dermatitis
**Suggested Intervention(s)** • Advise patients to use fragrance-free or anti-dandruff shampoo • Although part of the standard array change protocol, limit skin contact with alcohol-based products • Topical corticosteroids may be prescribed if inflammation is present (e.g., betamethasone, clobetasol, fluocinonide) • Identify cause and, if possible, reduce/eliminate		
**Case 3: TTFields AE – contact dermatitis**		
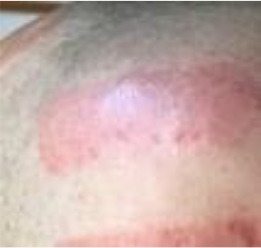	**Symptomatology**	**Potential Cause(s)**
	**Contact**	
	• Skin rash characterized by red, itching papules • May resemble a burn • Rash may present with red bumps that form moist, weeping blisters • Localized yet may be more diffuse than irritant type dermatitis	• Allergy to specific exogenous allergens, such as adhesive tape and/or hydrogel, that come into contact with the skin causing an inflammatory reaction
	**Irritant**	
	• Skin redness • Mild edema • Scaling • Rash that may be itchy or painful • Dermatitis restricted locally to the area of the irritant	• Non-specific inflammation caused by direct cellular damage upon contact with an inherently harmful substance to cells (e.g., chemical irritation from hydrogel, moisture, and/or alcohol)
**Suggested Intervention(s)** • Immediate removal of the irritant/allergen • Transducer array removal from irritation/allergen site • Topical corticosteroid (e.g., betamethasone, clobetasol, fluocinonide) application • Apply a barrier film • Consider trimming adhesive/surgilast if reaction exists to tape/adhesive • If blistering develops, cold, moist compress application (20 min; 3 times/day) is recommended • Consider systemic corticosteroids/treatment breaks if condition persists		
**Case 4: TTFields AE – erosion; and TTFields + bevacizumab AE – ulceration with hardware exposure**		
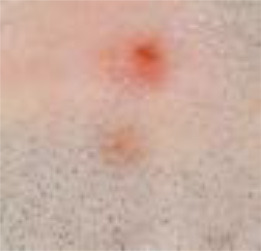	**Symptomatology** **Erosion**	**Potential Cause(s)**
	• Breakdown of the outer epidermal layer of skin • Skin discontinuity marked by incomplete loss of the epidermis • May present as a delineated moist or depressed lesion • Mild bleeding with pain or burning may be present • Typically, erosions do not result in scarring	• Mechanical trauma from shaving and/or array application/ removal • May develop from inflammation or maceration due to sweat, rupture of vesicles, bullae from infection, or epidermal necrosis
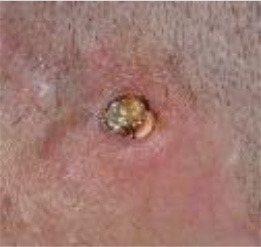	**Ulcer**	
	• Open scalp skin defects with potential for bleeding or oozing • Complete loss of epidermis and portions of the dermis, fat, or muscle, with increased risk of scarring • Pustules may develop when infected	• Ischemic injury and/or decreased perfusion produced by array pressure (especially in areas overlying scars, hardware, and prior radiation exposure
**Suggested Intervention(s)** • Transducer array removal from site of erosion/ulcer – consider re-placement to avoid hardware exposure • Wound dressing with gauzes, hydrogels, or hydrocolloids • Assess wound and treat with topical antibiotic (e.g., clindamycin, gentamicin) • Consider wound culture • Keep clear of excess discharge and dead skin (severe cases may require surgical debridement) • Return to clinic in 2 weeks; if condition persists, consider oral antibiotic/treatment break		
**Case 5: TTFields AE – dermatitis + infections**		
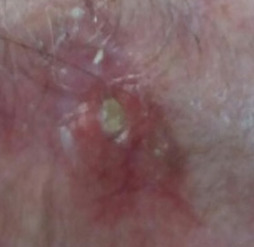	**Symptomatology**	**Potential Cause(s)**
	• Inflammation of skin or hair follicle (red pimple with hair in the center) • May have pus, itching, or burning	• Secondary bacterial infection • Ultimately, infection with or without pustules may occur when the skin is affected by pathogenic bacteria
**Suggested Intervention(s)** • Assess wound and treat with topical antibiotic (e.g., clindamycin or gentamicin) • Warm compresses with saltwater or Burow's solution (5% aluminum subacetate) • Take wound culture and potentially refer to dermatologist • Return to clinic in 2 weeks; if condition persists, consider oral antibiotic/treatment break		

## Management of Dermatologic Adverse Events

### Identification and Stratification of Risk Factors

Multiple factors increase the risk of developing dermatologic AEs in patients treated with TTFields (39). Prior craniotomies, especially those that necessitated scalp reconstruction, may increase risk (39, 41), and skin breakdown is more likely to occur from transducer array placement over surgical scar lines and surgical hardware from craniotomy repair. While surgical hardware is not an absolute contraindication for TTFields use, oncologists should consider this when planning array placement, and oncology nurses should discuss array and adhesive placement and avoidance of surgical hardware and scars with patients and caregivers.

Patients with pre-existing skin conditions or who previously developed contact dermatitis to any materials used on the arrays (adhesive and hydrogel) are at increased risk (39). Patients with hyperhidrosis (excessive sweating) have a higher complication rate due to the hydrophilic nature of the hydrogel disks in the arrays, which may liquefy upon exposure to sweat (39). Patients with persistent alopecia, which is a sign of depletion of epidermal stem cells in the follicular epidermis (42), may be more likely to develop dermatitis, and patients with a history of skin exposure to radiation (ultraviolet and/or ionizing) also have increased risk. Pre-existing acute or chronic effects from radiation therapy, such as dermatitis, scars, and fibrosis, may predispose the scalp to injury from the arrays (41). Radiation injury may also render skin less likely to recover from the subclinical alterations related to array placement, continued contact, and removal. Frequent array replacement beyond the recommended rate of twice per week may damage the outer layer of the epidermis by adhesive peeling and increased exposure of the dermis to irritants.

Patients being treated with systemic anticancer agents such as TMZ or bevacizumab, high doses of corticosteroids, or oral antibiotics such as penicillin or cephalosporin are also at increased risk of skin AEs (39). The anti-angiogenic agent bevacizumab may increase the likelihood of wound breakdown and delayed wound healing (41). Caution is therefore required when combining TTFields and bevacizumab outside a clinical trial. TMZ was found to cause neutropenia and thrombocytopenia, which increase the probability of developing secondary infections or severe bleeding, respectively (39). In cancer patients treated with TMZ, the greater incidence of high-risk AEs (e.g., hematological or gastrointestinal events) may contribute to skin AE marginalization when weighing treatment benefits against risks (e.g., treatment-related AEs) and burden of disease. Yet, TMZ treatment alone has been associated with skin AEs. In a recent phase 3 randomized trial, single-arm TMZ treatment of patients with newly diagnosed GBM demonstrated reported incidences of skin AEs that included alopecia (16%), exanthema/rashes (14%), and erythema (3%), as well as AE severity grades of 3 and 4 in some patients (~2%) (43). Hence, prudent monitoring of skin AEs and incorporating preventative measures is important when TTFields is combined with TMZ in patients with newly diagnosed GBM to best support patient care, as TMZ may contribute to the onset and/or exacerbation of skin AEs. Moreover, patients receiving dexamethasone, which can lead to dry and thinning skin, may also be more likely to develop skin AEs. Close monitoring is thus recommended.

Before and after TTFields therapy initiation, healthcare professionals should regularly examine and assess the scalp to detect, manage, and thus prevent worsening of any local skin irritation (44). Patients should have their scalp assessed for xerosis, dermatitis, or hyperhidrotic skin and treated accordingly prior to therapy initiation. The scalp and shoulders should be assessed for xerosis and healthcare professionals should advise patients to use fragrance-free or anti-seborrheic dermatitis (dandruff) shampoo if present and to avoid/limit skin contact with alcohol-based products. Topical corticosteroid solutions or lotions may be prescribed to reduce any inflammation present on the patient's scalp. Patients with grade 1 pruritus should be treated with topical corticosteroids (e.g., betamethasone, clobetasol, or fluocinonide), and oral gabapentin (100–600 mg 3 times a day as tolerated) or pregabalin (25–150 mg twice daily as tolerated; titrated up to 300 mg twice daily if necessary) should be considered for grade 2 pruritus. Hyperhidrosis may be treated with topical aluminum chloride antiperspirant or topical glycopyrrolate. In grade 2/3 cases, patients should be referred to a dermatologist for consideration of either oral glycopyrrolate or botulinum toxin injections. Increased skin health monitoring is especially recommended for patients with the risk factors described above.

### Prophylactic Interventions

**Figure 2:**
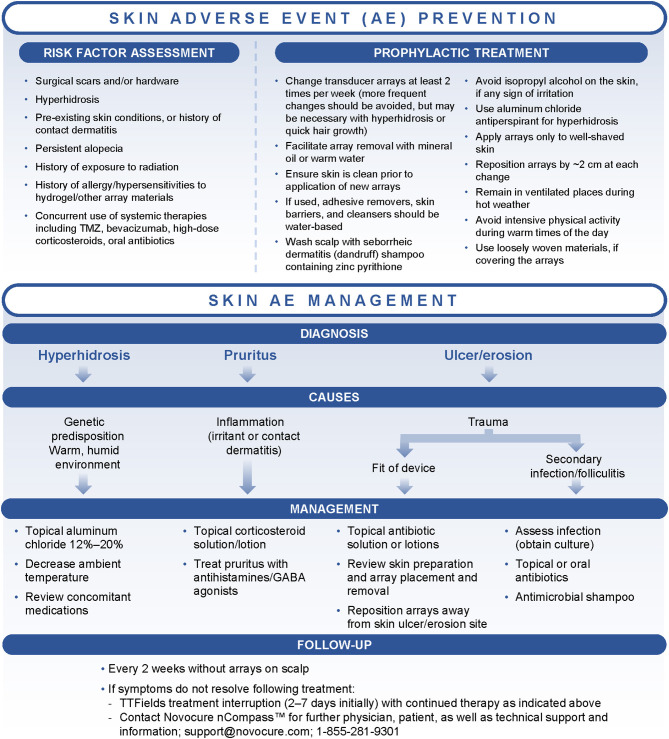
Treatment algorithms for the prevention and management of dermatologic adverse events (AEs) associated with Tumor Treating Fields (TTFields) application. GABA, gamma-aminobutyric acid; TMZ, temozolomide.

Early prophylactic interventions and good patient management strategies, including optimal shaving and array repositioning, may decrease the risk and severity of dermatologic AEs (Table 2; Figure 2) (41, 46). Shifting array position by ~2 cm when reapplying is an important preventive measure and helps minimize progression of current skin AEs (47). Arrays should be shifted in pairs, allowing them to continue to work in tandem. Ideally, arrays should be shifted back to their original position to ensure optimal targeting of the tumor bed (13). Arrays should be changed at least 2 times per week, approximately every 3 days, although some patients may benefit from more frequent replacement (e.g., with hyperhidrosis or quick hair growth).

**Table 2:** Prophylactic recommendations For patients treated with TTFields and caregivers (24, 39, 41, 45, 46).

**Prophylactic intervention**	**Recommendations For patients treated with TTFields and caregivers**
Optimal shaving and preparation of the scalp to maximize transducer-skin contact and minimize erosions and other factors increasing the risk of infection	• Perform proper hand washing before preparing the scalp for array application • Shave the scalp every time arrays are changed using gentle but firm circular motions – complete hair removal is required for optimal adhesion • Use a clean, electric razor to avoid cuts • Mineral (baby) oil may be applied before shaving to allow for cleansing of the skin and facilitate removal of bacteria and scale
Removal of natural oils and any moisture (sweat) from the scalp prior to array placement	• Wash the scalp with mild, fragrance-free shampoo (e.g., baby shampoo) or dandruff shampoo • If no skin irritation is present, wipe the scalp with a gauze or cotton ball soaked in first aid isopropyl alcohol (70%) • Ensure scalp is completely dry before array placement
Careful application and removal of transducer arrays is crucial to decrease the risk of cutaneous irritation	• Change arrays at least every 3–4 days, or more frequently if they become wet or loosen (e.g., excessive sweating during warmer weather or after intense physical activity) • Apply mineral (baby) oil to the scalp to gently remove arrays; slowly and gently peel back the arrays from the skin – pulling the skin or forceful rubbing of the scalp to remove adhesive can contribute to skin breakdown and irritation so should be avoided • Alternatively, the arrays may be removed in a hot shower by rubbing in a body wash containing coconut oil causing them to slide off the scalp • Evaluate the skin and scalp for signs of irritation with every array change, and notify your doctor or nurse if there are signs of irritation (taking a picture of the affected area is advised)
Regular array repositioning to minimize direct pressure to the scalp and ensure avoidance of surgical scar lines	• At each array change, shift array placement by ~2 cm, ensuring that pairs of arrays are moved together • Move arrays back to original position at subsequent change • Avoid ceramic disc placement immediately over scars or surgical screws • Wear breathable headwear to avoid overheating

Patient and caregiver education is an essential part of risk reduction, and oncology nurses play a critical role in educating patients and caregivers on how to integrate TTFields therapy into their daily life; this can subsequently improve acceptance and adherence (45, 48–50). Nurses should educate patients on what to expect at treatment initiation, emphasizing the importance of communicating any changes in skin condition to healthcare professionals as soon as possible. Follow-up is important, particularly in the first 2 months after treatment initiation. Awareness of prophylactic interventions (including skin preparation, array placement and removal, and monitoring for early signs of skin infection or inflammation) is key for patient and caregiver education to reduce risk of AEs (Table 2).

### Pharmacologic Interventions and Treatment Interruption

When dermatologic AEs develop, treatment decisions should be based on the type and severity of symptoms in line with the treatment algorithm (Figure 2). Additional recommendations are outlined in Table 1. In general, the primary interventions are topical corticosteroids (e.g., betamethasone, clobetasol, or fluocinonide) for irritant or contact dermatitis and topical antibiotics (e.g., clindamycin or gentamicin) for skin ulcers/erosions or infections, applied at the time of array changes and shifts (24, 39, 41). Instruct patients to apply a thin layer to the dry scalp after array removal and cleaning, allowing the agents to dry (24, 39, 45).

**Figure 3:**
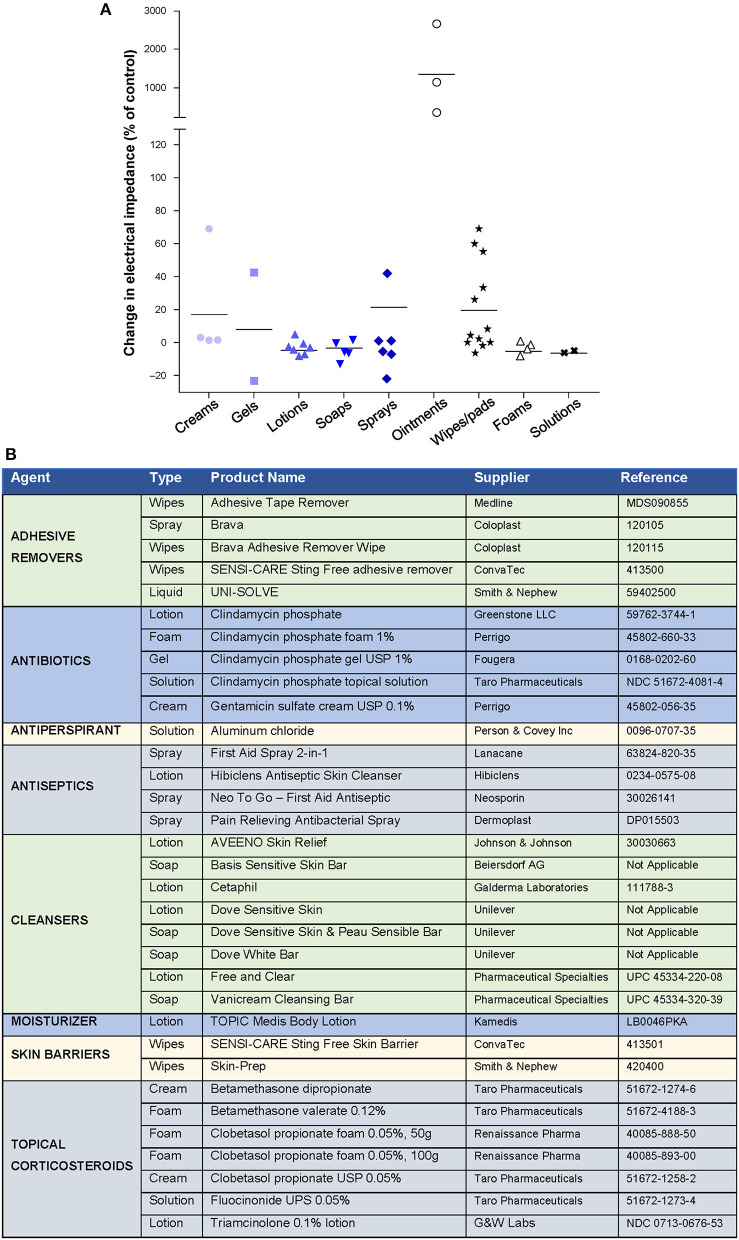
Effect of different skin care formulations on electrical impedance during Tumor Treating Fields (TTFields) application*. **(A)** Change in electrical impedance following application of skin care agent. **(B)** Products tested with minimal effects on electrical impedance. *Data Provided by Moshe Giladi, PhD, Preclinical Research, Novocure Ltd.

Topical agents (including antibiotics, corticosteroids, antiseptics, skin barriers, cleansers, moisturizers, and antiperspirants) are available in a variety of formulations that may affect TTFields efficacy by altering electrical impedance (51–53). In preclinical studies, water-based creams, gels, lotions, soaps, foams, wipes/pads, sprays, and solutions had minimal effects on electrical impedance (Figure 3A). Petroleum-based ointments led to the highest increases in impedance and are thus not recommended for use with TTFields. Products tested that were found to have minimal impact on impedance are listed in Figure 3B. Compatible treatments should be applied to the scalp in a thin layer to prevent impedance effects and optimize TTFields delivery.

Early detection of bacterial infections and appropriate treatment with topical or oral antibiotics is critical. Obtaining bacterial skin cultures prior to initiating antibiotic therapy is recommended to identify the causative microorganism(s) and ensure appropriate antimicrobial coverage (24). If uncertainty between an inflammatory or infectious AE exists, consultation with a dermatologist is recommended in addition to empirical therapy (41).

Treatment interruption may be required for intolerable grade 2 or worsening grade 3 dermatologic AEs (39). Interruption of array application for 2–7 days in addition to topical therapies is often sufficient for resolution of dermatologic AEs. Prolonged interruption may compromise treatment efficacy (39). Treatment interruption is also an option for recovery from a skin AE that does not improve, despite treatment (41). Although infrequent, skin ulcerations dictate array contact interruption until symptoms are grade 0/1 (45), which may require up to 14 days of treatment interruption.

### Practical Management in the Clinic

In addition to the recommendations above, the following practical advice based on clinical experience of TTFields-related dermatologic AE management may be adopted:

#### Patient Visits

- Ask patients direct questions (e.g., “Do you sweat a lot?”; “Is your scalp itchy?”).
- Patients are generally seen every 1–2 months for follow-up; increase follow-up to 2-week intervals if dermatologic AEs are present.
- Encourage patients to report any skin symptoms they experience to a healthcare professional to ensure timely management.
- Perform follow-up visits without transducer arrays on the patient's scalp to allow inspection of the skin. Array replacement should thus be synchronized with follow-up visits to minimize the frequency of array removal.
- If patients cannot be seen more frequently than every 2 months, 2-week follow-up can be performed remotely via phone or telemedicine.
- If available, use electronic medical record (EMR) systems to allow patients to send photos of their skin events to their treating team.
- Visiting nurse services should be used where available, especially in the absence of an array change partner or caregiver.

#### Patient Education to Prevent Dermatologic AEs

- Encourage patients to identify a transducer array change partner.
- Remind patients not to reuse the washcloth/towel that they use on their body to wash/dry their scalp.
- Advise patients to shift the array positions ~2 cm (0.75 inches) from the previous location when changing.
- For sensitive skin or hyperhidrosis, skin barrier products (Figure 3B) are recommended to impede moisture and protect against dermatitis, irritants, and minor injury.
- To help reduce shearing forces to the skin when removing arrays and adhesives, instruct patients to unplug the arrays and remove slowly after wetting the arrays in the shower, and/or to use adhesive removers (Figure 3B), mineral oils, or lubricating soaps for easy removal.
- Educate patients to adhere to recommended AE skin treatments to ensure continuous TTFields application.
- Emphasize the importance of avoiding placement of arrays over hardware and/or surgical scars.

## Discussion

TTFields are a novel anticancer treatment for GBM that involves placement of transducer arrays directly on the scalp. Since treatment is locoregional, the safety profile primarily includes local grade 1/2 dermatologic AEs beneath the arrays. Although the toxicity profile of TTFields is more favorable compared with most systemic therapies, treating oncologists and support staff should become familiar with the early identification and characterization of these unique AEs. Training on TTFields-related dermatologic AE prevention and management should be provided to all relevant staff, including oncologists, nursing/support staff, and dermatologists. In addition, training and education is required for patients and caregivers to ensure that appropriate steps are taken to maintain healthy skin and recognize when dermatologic events should be discussed with a healthcare professional.

Patient or physician concerns regarding dermatologic AEs should not be a barrier to starting or continuing TTFields therapy, since these are the only treatment-related AEs and are mild-to-moderate in severity in the vast majority of cases. Continuity of treatment is highly correlated with TTFields efficacy (26, 54). Physicians should thus consider referral to a dermatologist in patients with challenging skin conditions rather than discontinuing treatment. In addition, continuous use of prophylactic measures, combined with early identification and appropriate management of dermatologic AEs, can help patients maximize TTFields treatment time (54). Use of appropriate skin care strategies and medications to mitigate dermatologic AEs, removal of arrays to check scalp, and proactive skin AE prevention education, as well as balance of time on and off therapy, will help minimize treatment interruptions and maximize treatment adherence.

Management strategies for TTFields-associated dermatologic AEs are likely to evolve as adoption of the therapy increases and evidence from clinical trials and real-world settings accumulates; such as the recent, aforementioned, global post-marketing safety surveillance data from a large cohort (N=11,029) of patients with GBM, high-grade gliomas, and other brain cancers that provided real-world evidence that the safety profile of TTFields in the clinical practice setting remains consistent with published TTFields/GBM phase 3, clinical trial data (6, 31, 36). Clinical trials with TTFields in other solid malignancies are ongoing. TTFields (150 kHz) in combination with chemotherapy was recently approved by the FDA for first-line treatment of unresectable malignant pleural mesothelioma based on results from the STELLAR trial (55). Dermatologic AEs were also the main TTFields-related AE reported in that trial (68% in total, with 66% as grade 1/2). Our clinical practice recommendations may thus extend to patients with other malignancies receiving TTFields. Future studies, such as the randomized, double-blind PROTECT [NCT04469075] study (56), should help establish which treatments can best reduce skin AEs in patients with GBM.

## Conclusions

The recommendations outlined here represent our current understanding of the optimal prevention and management of TTFields-related dermatologic AEs. Application of these recommendations by healthcare professionals should help patients to minimize dermatologic AEs, improve patient quality-of-life, support adherence to therapy, and potentially improve treatment outcomes.

## Author Contributions

All authors contributed to the concept of this review article, wrote, edited, and reviewed the drafts, and approved the final manuscript.

## Conflict of Interest

ML reported a consulting role for Novocure, and funding in part by the national cancer institute center support Grant P30-CA008748. MA reported honoraria for consulting role and/or speaking engagements from Abbvie, Adgero, AstraZeneca, Boehringer Ingelheim, Bristol Myers Squibb, Biogen, Eisai, Eli Lilly, Genentech, ImClone, Innovaderm, OnQuality, Novocure, Therakos, and Xoma, and served as a Principal Investigator for Biogen, Veloce, Xoma, Hana Biosciences, InflamRx, Novartis, AnaptysBio, Boehringer Ingelheim, Lutris, OnQuality, ChemoCentryx, Eli Lilly, Abbvie, and XBiotech. MB reported a consulting role for Novocure. FI reported consulting and advisory board roles for Merck, Novocure, Regeneron, AbbVie, Alexion, Guidepoint, and Tocagen, and received research support (institutional) from Novocure, Bristol-Myers Squibb, Celldex, Northwest Biotherapeutics, Stemline, Regeneron, Incyte, Immunocellular Therapeutics, Merck, and Forma. SJ reported a consulting role for Novocure. reported advisory board role and speaker bureau membership for Novocure. MS reported a consulting role and speaker bureau membership for Novocure. JS reported a consulting role and speaker bureau membership for Novocure. MG reported personal fees and a consulting role for Bayer, Medac, Novocure, Merck, Kyowa Kirin, Roche, Novartis, AbbVie, Daiichi Sankyo, Amgen, and Janssen. The remaining author declares that the research was conducted in the absence of any commercial or financial relationships that could be construed as a potential conflict of interest.
